# Distribution and Difference of Gastrointestinal Flora in Sheep with Different Body Mass Index

**DOI:** 10.3390/ani12070880

**Published:** 2022-03-30

**Authors:** Jiangbo Cheng, Weimin Wang, Deyin Zhang, Yukun Zhang, Qizhi Song, Xiaolong Li, Yuan Zhao, Dan Xu, Liming Zhao, Wenxin Li, Jianghui Wang, Bubo Zhou, Changchun Lin, Xiaoxue Zhang

**Affiliations:** 1 College of Animal Science and Technology, Gansu Agricultural University, Lanzhou 730070, China; 15117098920@163.com (J.C.); wangwm@gsau.edu.cn (W.W.); greenday.zyk@foxmail.com (Y.Z.); songqz0613@163.com (Q.S.); lixllil@163.com (X.L.); zhaoyuan_10@163.com (Y.Z.); xud@st.gsau.edu.cn (D.X.); zlmfxy1807285865@163.com (L.Z.); liwx@st.gsau.edu.cn (W.L.); wangjianghui2022@163.com (J.W.); zhoubb@st.gsau.edu.cn (B.Z.); lincc@st.gsau.edu.cn (C.L.); 2 The State Key Laboratory of Grassland Agro-Ecosystems, College of Pastoral Agriculture Science and Technology, Lanzhou University, Lanzhou 730020, China; gsauzdy@163.com

**Keywords:** sheep, body mass index, fat deposition, functional prediction, gastrointestinal microorganisms

## Abstract

**Simple Summary:**

Fat deposition capacity greatly impacts the production capacity of sheep. The production of high amounts of fat affects the economic benefits of animals. Gastrointestinal microorganisms play an important role in the characteristics of host fat deposition. This study compares differences in gastrointestinal microorganisms in sheep with different body mass indices. Results showed that there were different microflora compositions among different groups. This provides a new idea for the regulation of fat deposition traits in sheep.

**Abstract:**

Fat deposition is the key factor affecting the efficiency of animal husbandry production. There are many factors affecting fat deposition, in which the gastrointestinal microbiota plays an important role. Therefore, the body mass index (BMI) was introduced into the evaluation of sheep fat deposition, and the different microbiota and functional pathways of the sheep gastrointestinal tract in different BMI groups were analyzed. We selected 5% of individuals with the highest and lowest BMI from a feed test population (357 in whole group). Microorganisms in 10 sites of the gastrointestinal tract in 36 individuals (18 in each group) were evaluated by 16S rRNA V3–V4 region sequencing. There were differences (*p* < 0.05) in fat deposition traits between different BMI groups. In the 10 parts of the gastrointestinal tract, the diversity and richness of cecal microflora in the high-BMI group were higher than those in low-BMI Hu sheep (*p* < 0.05). Principal coordinate analysis (PCoA) showed that there was separation of the cecum between groups, and there were differences in the cecal microbial community. Linear discriminant analysis effect size (LEfSe) showed that most biomarkers were in the cecum. On the basis of an indepth study of cecal microorganisms, 26 different bacterial genera were obtained (*p* < 0.05). Correlation analysis between them and the characteristics of fat deposition in sheep showed that *Colidextribacter*, *Alloprevotella*, and *Succenivibrio* were positively correlated with fat deposition, while *Lachnospiraceae_ND3007_Group* was negatively correlated (*p* < 0.05). The above results show that the cecum may be an important part leading to the difference of BMI in sheep, and its microorganisms may affect the level of fat deposition.

## 1. Introduction

Sheep (*Ovis aries*) belong to the Artiodactyla cattle family, which is a major economic livestock species. They digest and absorb nutrients through the gastrointestinal tract of ruminants. Sheep is one of the earliest domesticated livestock, widely distributed, especially in China, and there are many local subspecies [1]. Mutton has high nutritional value and low cholesterol content, which is popular on the market [2]. The fat deposition characteristic of sheep is one of the important indices to evaluate its meat production performance. Therefore, excessive fat deposition negatively affects the quality and economic benefits of mutton [3].

In sheep, fat deposition is a complex trait, which is composed of, for example, back, mesenteric, perirenal, and tail fat. Body mass index (BMI) can be utilized to reflect the body’s fat and thin degree, and it is also a predictor of energy reserve [4]. By using the ratio of body weight to body height squared to evaluate the degree of obesity, it is not easy to be affected by a change in height [5]. Compared with fat content measured by expensive instruments, the body mass index calculated by using simple body size data is simpler and more economical to reflect the fat content of the body [6,7]. However, correlation between BMI and fat deposition traits in sheep has not been reported. 

Many studies have shown that BMI is largely affected by heredity. Through genomewide association analysis, more than 300 single-nucleotide polymorphisms were identified to be associated with obesity traits such as BMI [8]. Moreover, microorganisms in the body affect BMI. Gastrointestinal microorganisms play an important role in the normal development, digestion, and metabolism of the host. Studies showed that it has an effect on human obesity and BMI [9,10,11]. Yang et al. reported that intestinal microbiota had an effect on fat deposition in sheep tail, and *Lachnospiraceae* and *Akkermansia* may be the key microbiota [12]. Lin et al. believe that microorganisms act on host physiology through metabolites, resulting in mitochondrial fragmentation and lipid accumulation [13].

In ruminants, most researchers pay more attention to the rumen [14,15], and less to the intestinal microflora and its impact on the host phenotype. However, the digestive tract of ruminants is a complex system in which different parts play different roles, and microorganisms have spatial heterogeneity. Wang et al. showed that microorganisms change along the gastrointestinal tract of sheep, in which the community composition of stomach, and the small and large intestines is obviously separated [16]. Xie et al. studied the microbial composition of 10 gastrointestinal parts of 7 ruminants and reached a consistent conclusion [17]. Therefore, microorganisms in different gastrointestinal tracts may play different functions.

In this study, the composition and functional changes of bacteria in 10 parts of the gastrointestinal tract of Hu sheep with different BMIs were compared and analyzed, aiming to find the key parts and microorganisms that affect the BMI of sheep, so as to determine the key differences of gastrointestinal microbes and reveal the microbiota related to fat deposition in sheep.

## 2. Materials and Methods

### 2.1. Animals and Groups

The experiment sheep (357 male Hu sheep) were purchased from a commercial sheep farm (Gansu Zhongsheng Huamei Sheep Industry Development Co. Ltd. In Qingyang City, Gansu Province, China). Immunization was conducted according to standardized procedures before weaning, and the sheep were weaned when they reached 56 days of age. All lambs were kept indoors in separate enclosures (0.8 × 1 m) until they were 180 days old. In short, all lambs were exposed to a period of adaptation of 14 days; during this period, the dietary proportion of pellet feed (Gansu Sanyangjinyuan Husbandry Co. Ltd. In Jinchang City, Gansu Province, China) was gradually increased by 7.1% every day, and the silage alfalfa proportion was simultaneously decreased until the pellet feed proportion had become 100%. The pellet feed consisted of 27% barley straw, 44% corn, 2.2% soybean meal, 2.60% rapeseed meal, 4.20% cottonseed meal, and 20% concentrate, containing 16.28% crude protein, 28.48% starch, 36.54% neutral detergent fiber, 14.12% acid detergent fiber, 0.60% calcium, and 0.30% phosphorus. All animals had ad libitum access to water and pellet feed. They were weighed using a calibrated electronic scale before feeding in the morning. Growth traits such as body weight and height, body length, and cannon circumference at 180 days old were recorded. Feeding methods and the environment were kept consistent at all times during the experiment.

BMI is calculated as body weight/body length^2^. Mean ± 3× standard deviation was used to eliminate abnormal BMI values in the test population. The population was sorted according to BMI, and 5% of the highest and lowest BMI in the test population were selected and divided into two groups. Low-BMI individuals were Group 1, and high-BMI individuals were Group 2.

### 2.2. Sample Collection and Character Determination

After 180 days of age determination, 36 sheep in the 2 experimental groups were slaughtered by carotid bloodletting, and the intact gastrointestinal tract of the experimental animals was taken out and ligated at the boundaries of different parts to prevent contamination caused by the flow of the contents. Content samples were collected at corresponding locations in 10 sites: rumen (L), reticulum (W), omasum (B), abomasum (Z1), duodenum (S), jejunum (K), ileum (H), cecum (M), colon (J) and rectum (Z2). Samples were temporarily stored in liquid nitrogen (−196 °C). After the slaughter test, samples were transported back to the laboratory and transferred to a −80 °C ultralow-temperature refrigerator for storage.

The fat deposition characteristics of sheep were determined during the slaughter test. Backfat thickness: fat thickness of the posterior edge of scapula, the rib end, and the front edge of hip tubercle was measured, and the average value was taken [18]. Carcass fat content (GR) [19]: tissue thickness between the 12th and 13th ribs and 11 cm from the midline of the spine was measured. Perirenal, mesenteric, and tail fat of the test individual was taken out, weighed with an electronic scale, and the relative weight of fat was calculated with premortem live weight.

### 2.3. DNA Extraction and Amplification

Cetyltrimethylammonium Bromide (CTAB) was used to extract DNA from the samples. Agarose gel electrophoresis was used to detect the purity and concentration of DNA. Some samples were diluted to 1 ng/µL with sterile water (TransGen Biotech, Beijing, China). The remaining DNA samples were stored at −20 °C. 

The extracted DNA was used as a template for PCR amplification, and primers were 314F (CCTAYGGGRBGCASCAG) and 806R (GGACTACNNGGGTATCTAAT). The amplified region was the V3–V4 region of microbial 16S ribosomal RNA. The PCR used a 30 μL system: 15 μL Phusion^®^ High-Fidelity PCR Master Mix (New England Biolabs), 0.2 μm upstream and downstream primers, 10 ng template DNA and 2 μL sterile water. Cycling conditions were as follows: 1 min at 98 °C; 10 s at 98 °C, 30 s at 50 °C, and 30 s at 72 °C for 30 cycles; lastly, 5 min at 72 °C. The same volume of 1X loading buffer (contained SYB green) was mixed with PCR products and electrophoresis was operated on 2% agarose gel for detection. Samples with a bright main strip in the range of 400–450 bp were chosen for further experiments. PCR products was mixed in equidensity ratios. Then, the mixture of PCR products was purified with GeneJET Gel Extraction Kit (Thermo Scientific. Waltham, MA, USA). 

### 2.4. Library Construction and Data Processing

The library was constructed by using the library building kit (TruSeq DNA PCR-Free Library Preparation Kit, Illumina, San Diego, CA, USA). Library quality was assessed on a Qubit @ 2.0 Fluorometer (Thermo Scientific) and Agilent Bioanalyzer 2100 system. Lastly, the library was sequenced on an Illumina NovaSeq platform, and 250 bp paired-end reads were generated.

Sequenced DNA fragments were paired-end reads using FLASH (version 1.2.7) [20]. Paired-end reads were assigned to each sample according to the unique barcodes. Sequences were analyzed using QIIME [21] software (version 1.9.1) (Quantitative Insights into Microbial Ecology), and inhouse Perl scripts were used to analyze alpha (within-samples) and beta (among-samples) diversity. First, reads were filtered with QIIME quality filters. Then, we used pick_de_novo_otus.py to choose operational taxonomic units (OTUs) by producing an OTU table. Sequences with ≥97% similarity were assigned to the same OTUs. We chose representative sequences for each OTU and used the RDP classifier [22] to annotate taxonomic information for each representative sequence. In order to compute alpha diversity, we rarified the OTU table and calculated three metrics: Chao1, which estimates species abundance; Observed Species, which estimates the number of unique OTUs found in each sample; and the Shannon index. Rarefaction curves were generated on the basis of these three metrics. QIIME (Version 1.9.1) calculates unweighted UniFrac, which are phylogenetic measures of beta diversity. We used unweighted UniFrac for principal coordinate analysis (PCoA) and unweighted pair group method with arithmetic mean (UPGMA) clustering. OTUs were compared and annotated with the SILVA138 database with the Mothur method [23]. To mine deeper data of the microbial diversity of the differences between samples, significance tests were conducted with some statistical analytical methods, including the t-test, LEfSe, rank sum test, MetaStat, and MRPP. Results were visualized using R software (Version 2.15.3).

Tax4Fun function prediction is achieved by the nearest-neighbor method based on minimal 16S rRNA sequence similarity. The specific method is to extract the KEGG database prokaryotic whole genome 16S rRNA gene sequence and use the BLASTN algorithm to align it to the SILVA SSU Ref NR database (BLAST bitscore > 1500) to establish a correlation matrix, and map the prokaryotic genomewide functional information of the KEGG database annotated by the UProC and PAUDA methods to the SILVA database to realize the functional annotation of the SILVA database. Sequenced samples were clustered with the SILVA database sequence as the reference sequence to cluster OTUs and obtain functional annotation information.

## 3. Results

### 3.1. Baseline Characteristics of Test Population

We used Spearman correlation to analyze the correlation between sheep BMI and various fat deposition traits, as shown in Table 1. Results showed that BMI was significantly correlated with various fat deposition traits (*p* < 0.01), indicating that BMI can effectively assess the level of fat deposition in sheep.

In total, 36 Hu sheep were enrolled in the present cross-sectional study. The trait characteristics of the test population are shown in Table 2. When *p*-value < 0.05, the difference was statistically significant. As shown in Table 2, the fat deposition traits in the high-BMI group were higher than those in the low-BMI Hu sheep group except for the relative weight of tail fat (*p* < 0.05). The difference in body length between the two groups was not statistically significant (*p* > 0.05).

### 3.2. Sequencing Data Overview

Samples were taken from the digestive tract of 36 Hu sheep divided into high- and low-BMI groups. We successfully amplified the 16S rRNA sequence from contents collected from 10 different gastrointestinal regions of Hu sheep. All 339 result samples were sequenced, and 30,842,807 raw tags were generated after splicing (Table S1). After filtering the low-quality sequences and chimeras, generated clean tags and effective tags are represented in Supplementary File S1. The average length of each sequence was 414 bp. A total of 20,928,585 sequences were used for follow-up study after quality control. These sequences were clustered into 7788 operational taxonomic units (OTUs) using the MOTHUR method. In order to confirm whether the sequencing depth and sample size could meet the analytical requirements, we conducted dilution and species accumulation analysis. With the increase in sequencing depth and sample size, the dilution curve and species accumulation curve tended to be flat, indicating that this condition met the analytical requirements (Figure S1).

### 3.3. Diversity Analysis

In order to study the microbial community composition in different areas of the gastrointestinal tract of two groups of sheep, we evaluated the alpha diversity differences in ten test areas (Table S2). Results showed differences in the alpha diversity of digestive tract microbes among different BMI Hu sheep groups (*p* < 0.05). The cecal microbial Shannon index and Chao1 index were different between the two groups (*p* < 0.05), and the cecal microbial diversity and richness of high BMI Hu sheep were higher (*p* < 0.05).

**Figure 1:**
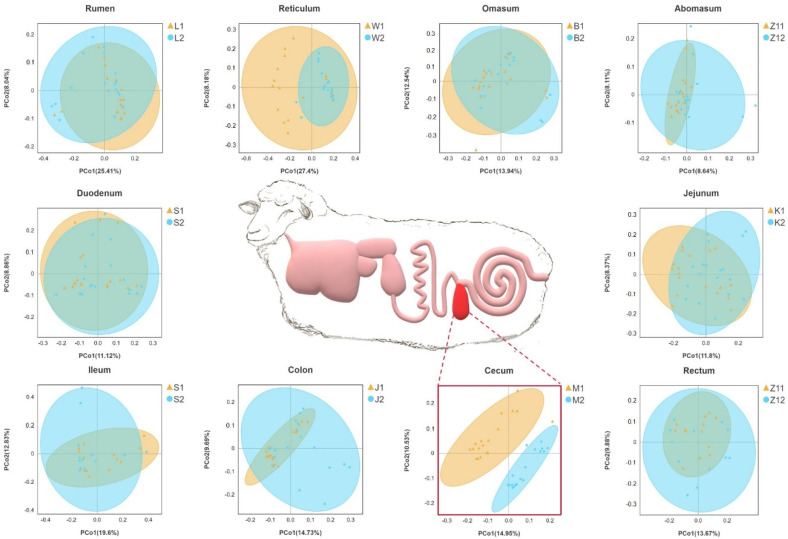
Gastrointestinal structure of Hu sheep. Principal coordinate analysis was performed on the basis of unweighted UniFrac distances to identify differences in microbial community structure across taxa. Sheep with high BMI showed round points, and sheep with low BMI showed triangular points.

On the basis of the phylogenetic relationship between OTUs, UniFrac distance was calculated. Principal coordinate analysis (PCoA) based on unweighted UniFrac distance showed that the cecum were significantly separated between the two groups, and other parts overlapped between the two groups (Figure 1).

We also used MRPP analysis to evaluate differences in the community composition between the two groups (Table 3). In the microbial community structure of the rumen (A = −0.0005) and duodenum (A = −0.0032) between the two groups, difference within a group was greater than that between the groups. There were significant differences in the community structure of the reticulum, flap stomach, cecum, colon, and rectum between the two groups (*p* < 0.05), which confirmed the PCoA results.

### 3.4. Analysis of the Gastrointestinal Tract Microbiota Composition

**Figure 2:**
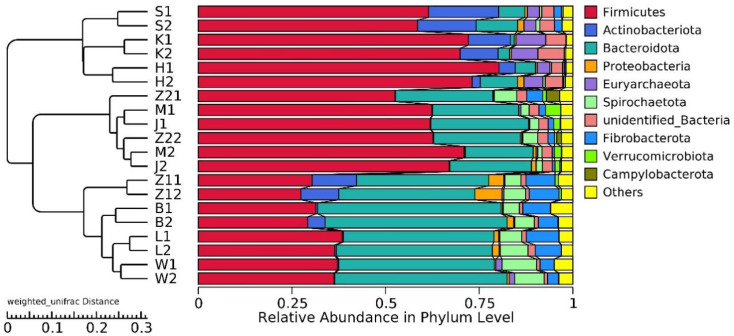
Histogram of gastrointestinal relative abundance in different groups at phylum level, and UPGMA clustering tree based on weighted UniFrac distance.

20,928,585 effective tags were annotated by the 16S Silva database, which was divided into 47 phyla, 115 classes, 248 orders, 358 families, and 634 genera. Unallocated taxa accounted for 2.94% of the total OTUs. At the phylum level, the abundance of *Firmicutes* and *Bacteroidetes* in the gastrointestinal tract of Hu sheep in high- and low-BMI groups was higher. *Actinobacteriota* also had a high abundance in the duodenum, jejunum, and ileum (Figure 2).

**Figure 3:**
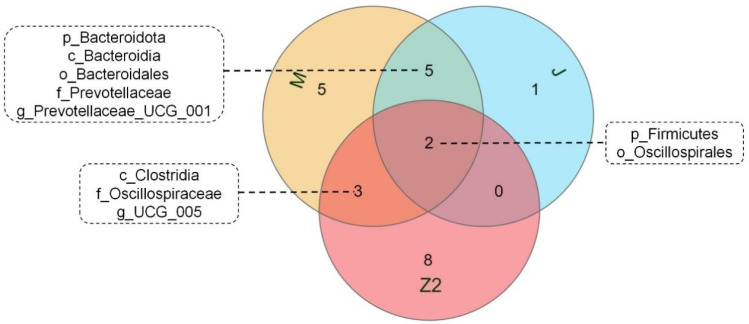
Venn diagram of differential biomarkers based on LEfSe analysis in cecum, colon, and rectum.

We performed LEfSe analysis on high- and low-BMI Hu sheep groups using LDA score = 4 (Figure S2). There were biomarkers in gastrointestinal microorganisms among different groups that were statistically different. Among the 10 sites of microbes in the 2 groups of sheep, the large intestine (cecum, colon, rectum) had the most differential biomarkers, and differential biomarkers were similar in the three sites. We used a Venn diagram to analyze cecum and colorectal biomarkers, in which *Firmicutes* and *Oscillospirales* were common, and the cecum contained the most biomarkers (Figure 3). *Firmicutes*, *Clostridia*, *Oscillospirales*, *Oscillospiraceae,* and *UCG_005* are biomarkers in the cecum of high-BMI Hu sheep, while *Verrucomicrobiota*, *Verrucomicrobiae*, *Verrucomicrobiales*, *Akkermansiaceae, Akkermansia*, *Bacteroidota, Bacteroidia*, *Bacteroidales*, *Prevotellaceae,* and *Prevotellaceae_UCG_001* are biomarkers in the cecum of low-BMI Hu sheep.

### 3.5. Cecal Microorganisms Affect Fat Deposition

**Figure 4:**
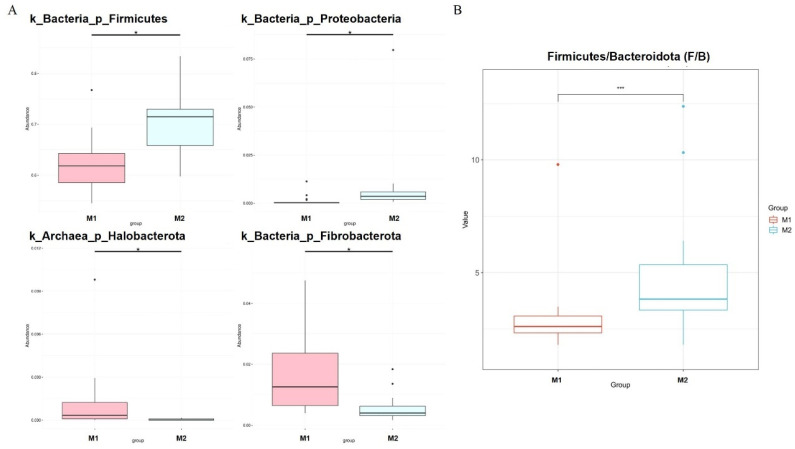
Dfferences in microbes at cecal phylum level in Hu sheep with different BMI. * means *p* < 0.05, *** means *p* < 0.001. (**A**) Phylum level; (**B**) difference in ratio of *Firmicutes* to *Bacteroidetes*.

We used the Metastat [24] method to deeply study microbiota with significant differences in the cecum between the two groups (Figure 4A). *Fibrobacterota* and *Halobacterota* numbers in the cecum of low-BMI Hu sheep were significantly higher than those of high-BMI Hu sheep (*p* < 0.05), *Firmicutes* and *Proteobacteria* were significantly lower than those of high BMI Hu sheep (*p* < 0.05). The abundance ratio of *Firmicutes* to *Bacteroidetes* in the high-BMI Hu sheep population was significantly higher than that in the low-BMI Hu sheep population (*p* < 0.05) (Figure 4B). 

At the genus level (Table 4), 12 bacterial genera such as *Saccharofermentans*, *Prevotella* and *Erysipelotrichaceae* in the cecum of low-BMI Hu sheep were significantly higher than those of the high-BMI Hu sheep (*p* < 0.05). The numbers of 14 bacterial genera, such as *Oscillospiraceae_UCG-005*, *Colidextribacter* and *Agathobacter,* were significantly lower than those in the cecum of high-BMI Hu sheep (*p* < 0.05).

**Figure 5:**
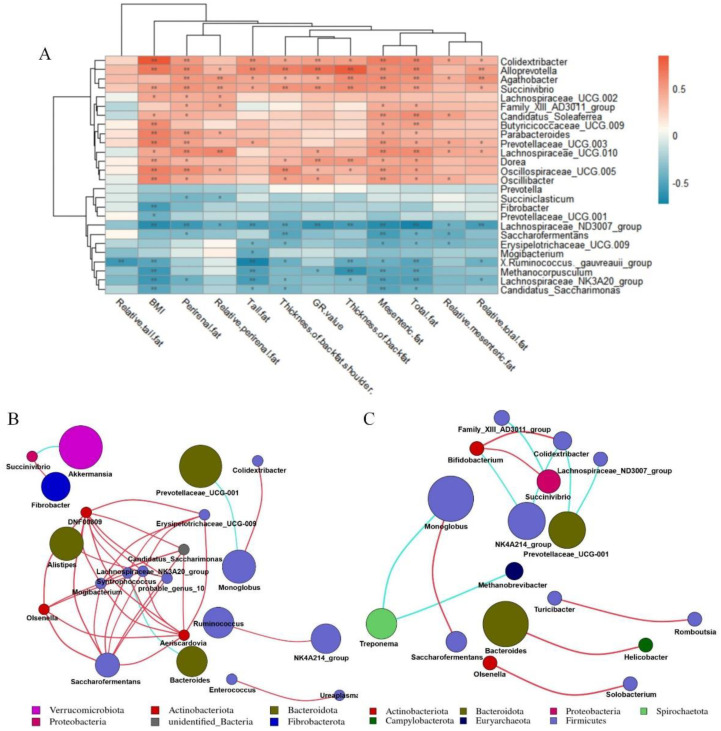
Correlation analysis. Correlation network analysis diagram constructed according to annotation results. Correlation network deduced from the first 50 genera. Each point represents a genus, and the size of the point is directly proportional to the relative abundance of the genus. Lines between points indicate correlation relationship between genera: red is positive correlation, blue is negative correlation, and only lines with correlation greater than 0.6 are displayed. (**A**) Correlation analysis between cecal differential microorganisms and fat deposition traits of Hu sheep. *, *p* < 0.05; **, *p* < 0.01. (**B**) Correlation network diagram of genus-level microorganisms in cecum of low-BMI Hu sheep group. (**C**) Correlation network diagram of genus-level microorganisms in cecum of high-BMI Hu sheep group.

We took 26 different bacterial genera in different groups of the cecum as the research object to analyze the correlation between different bacterial genera and fat deposition traits of Hu sheep (Figure 5A). The different bacterial genera in the cecum were related to the characteristics of fat deposition in Hu sheep, including *Oscillospiraceae_UCG-005, Butyricicoccaceae_UCG-009, Alloprevotella, Dorea, Succinivibrio, Prevotellaceae_UCG-003, Colidextribacter, Parabacteroides, Oscillibacter* were all positively correlated with BMI (*p* < 0.01). Moreover, *Candidatus_Saccharimonas*, *Fibrobacter, Lachnospiraceae_NK3A20_group, Lachnospiraceae_ND3007_group, Methanocorpusculum, [Ruminococcus]_gauvreauii_group* were negatively correlated with BMI (*p* < 0.01). While *Colidextribacter*, *Alloprevotella*, *Succenivibrio* *and Lachnospiraceae_ND3007_Group* were associated with more than 80% of the fat deposition traits (*p* < 0.05).

On the basis of the Spearman correlation coefficient, the correlation network of the top 50 genera of cecum of Hu sheep with different BMI was analyzed. *Bacteroides* was negatively correlated with *Syntropococcus* in the cecum samples of low-BMI Hu sheep, while *Syntropococcus* and *Firmicutes* were positively correlated with the genera of multiple *Firmicutes* (Figure 5B). However, this was not observed in the genus level network of cecum of Hu sheep with high BMI (Figure 5C).

### 3.6. Functional Prediction Analysis

**Figure 6:**
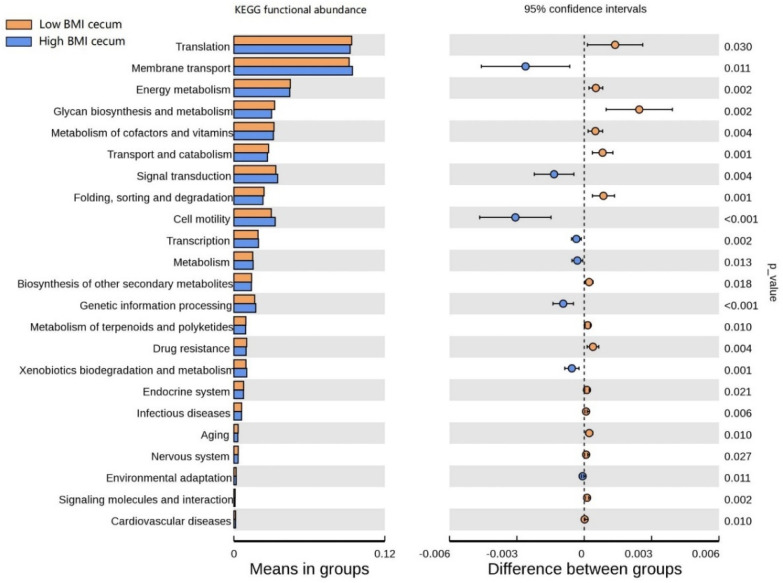
Differences of known functional pathways were compared. Tax4Fun used to evaluate functional contribution of cecal microorganisms.

The functional clustering heat map showed that similar functional pathways existed in the stomach, and small and large intestine groups. The cecum, colon, and rectum showed higher clustering in the same group (Figure S3). In order to determine the functional pathways related to BMI, gastrointestinal tract microorganisms of Hu sheep with high and low BMI were compared. The functional pathway of cecal flora was evaluated using Tax4Fun. At Level 1, the cecum was composed of the most differential functional pathways, mainly in metabolism, human diseases, and systems (Figure S4). Therefore, the cecal functional pathways were analyzed at Level 2 (Figure 6), and 23 differential pathways were obtained. Among them, the enrichment degree of low BMI Hu sheep in translation, energy metabolism, glycan biosynthesis, and metabolism was significantly higher than that of the high-BMI Hu sheep (*p* < 0.05). Eight pathways, such as membrane transport, signal transmission, and cell mobility, were significantly enriched in the high-BMI Hu sheep population (*p* < 0.05). These findings clearly show that, when BMI is different, there are great differences in the functional pathways of cecal microbial enrichment.

## 4. Discussion

A number of studies were conducted in humans to show the degree of obesity through BMI [25,26]. To our knowledge, it is rare to use BMI to evaluate sheep fat deposition. Previous studies on the sheep gastrointestinal tract mainly focused on rumen samples [27]. However, there is spatial heterogeneity in microbial colonization in the digestive tract. The evaluation of BMI on fat deposition is carried out in humans. Results show that BMI can effectively judge the content of subcutaneous adipose tissue. Its description of body fat is similar to waist circumference, waist body ratio, and other indicators, which can effectively reflect the proportion of fat content [28,29].

Similar results were obtained in the study of the sheep cohort. This study found that most correlation of fat deposition in the high-BMI group was significantly higher than that in the low-BMI group, which showed that high-BMI Hu sheep had higher fat deposition ability. A cross-sectional correlation study showed that the higher the height is, the higher the risk of subsequent obesity [30]. We used body length instead of height to calculate BMI, and there was no significant difference in body length between different groups, which reduces the impact of body length on BMI calculation, verifying our previous speculation that BMI can reflect the obesity level of Hu sheep.

Along the gastrointestinal tract of sheep, microbial community structure and composition changed. There were significant differences in the alpha diversity index between the high- and low-BMI groups in the reticulum and cecum, including the Shannon and Chao1 indices. The diversity index showed the same trend in the cecum, which was higher in the high-BMI group. Our results showed that the cecal microorganisms of high-BMI Hu sheep showed higher diversity and richness. Our results are consistent with those of previous studies, which found that the intestinal alpha diversity of overweight people is higher than that of normal-weight people, and the bacterial diversity of obese subjects is higher [30,31]. Obesity leads to the dysbiosis of the gut microbiota, with increased types and abundances of obesity-related microbiota [10,32]. Principal coordinate analysis (PCoA) based on UniFrac distance showed a separation of the cecum and colon between different groups and aggregation within the group. This shows that there are differences in the microbial community composition of cecum and colon samples in different groups. We verified this with the MRPP method, and confirmed that there are significant differences between cecum and colon in different groups. These results were consistent with those of other species that found that there was separation in the principal coordinate analysis of cecal samples of normal and obese mice [33].

According to our findings, there are differences in the relative abundance of microorganisms along the gastrointestinal tract of Hu sheep. These results are consistent with previous research results in humans [34], rats [35] and donkeys [36]. Similar to previous reports, the duodenum and jejunum are mainly composed of *Firmicutes* and *Actinobacteriota* [37], while the rest of the gastrointestinal tract is composed of *Firmicutes* and *Bacteroidetes*. However, the relative abundance of *Bacteroidetes* is the highest in rumen, reticulum, omasum and abomasum [38], while the relative abundance of *Firmicutes* is the highest in ileum, cecum, colon and rectum [39]. Combined with the UPGMA cluster tree, the stomach, and small and large intestines form three clusters. Only clusters between the high- and low-BMI groups in the large intestine had differences, and the enrichment of functional pathways also showed consistent results. This may be related to the fermentation degree of the large intestine because microorganisms provide nutrition and energy to the host by fermenting undigested components, but there may be a risk of obesity [40,41].

Combined with LEfSe analysis, biomarkers in different parts of the gastrointestinal tract of Hu sheep were determined. *Firmicutes* and *Bacteroidetes* are biomarkers in the cecum, colon, and rectum. Many previous studies reported that obesity is related to an increase in *Firmicutes* and a decrease in *Bacteroidetes* [42,43]. *Akkermansiaceae* in the cecum was observed to be negatively correlated with overweight and obesity [44]. These are consistent with our results. In addition, the cecum comprises the most biomarkers in the gastrointestinal tract of different groups, suggesting that the cecum may be an important site related to BMI in the gastrointestinal tract of sheep.

In order to determine the key microbiota related to BMI, differences in cecal microorganisms in different groups were analyzed. We obtained a phylum consistent with LEfSe results, namely, *Firmicutes*. In high-BMI Hu sheep, the relative abundance ratio of *Firmicutes* and *Bacteroidetes* was significantly higher than that of the low-BMI Hu sheep. This is consistent with results obtained in other reports in both humans and rats [45,46]. The increase in *Proteobacteria* was observed to be related to obesity and intestinal microbiota imbalance in animals, and the level of *Proteobacteria* decreased in the experiment of reversing obesity [47,48].

In this study, we identified 19 bacterial genera significantly related to BMI, of which *Colidextribacter*, *Alloprevotella* and *Succenivibrio* were significantly positively correlated with more than 80% of fat deposition traits, while *Lachnospiraceae_ND3007_Group* showed significantly negatively correlated. In this study, negative correlation between *Lachnospiraceae_ND3007_Group* with BMI and fat deposition was identified for the first time. In previous reports, *Colidextribacter* and *Alloprevotella* were positively correlated with hyperlipidemia in rats and daily gain in pigs, respectively [49,50], and we found them in the cecum of high-BMI Hu sheep. In particular, *Saccharivibrio* belonging to *Proteobacteria* presented high relative abundance in the cecum of high-BMI Hu sheep. *Succinivibrio* can ferment to produce a variety of sugars. The main metabolic end products are acetic acid and succinic acid, and small amounts of formic acid and lactic acid. A high abundance of *Succinivibrio* produces more acetic acid, and acetic acid promotes lipid synthesis; we can speculate that a high concentration of acetic acid is absorbed by the intestinal epithelium, which improves the efficiency of fat deposition [51]. In conclusion, the four bacterial genera above may be key bacterial genera related to the host BMI, which suggests the need for further research.

Changes in intestinal microorganisms may reshape intestinal barrier function, host metabolism, and signaling pathways [52]. Studies showed that transplanting gut microbiota from normal mice into sterile mice increases body fat in sterile mice without increasing food intake [53]. We, therefore, analyzed the microbial function of the sheep gastrointestinal tract. The cecum is composed of the most differential functional pathways. Membrane transport and cell motility are highly enriched in the cecum of Hu sheep with high BMI, which indicates that microorganisms affect the epithelial cells of the gastrointestinal tract and improve energy absorption. In contrast, energy metabolism, transport and catabolism, folding sorting, and the degradation pathway are enriched in the cecum of low-BMI Hu sheep, suggesting that microorganisms may reduce fat deposition by accelerating the rate of metabolism. However, further research is needed to describe the specific functional pathway differences of different microorganisms. At present, analysis of functional differences is predictive.

## 5. Conclusions

This study introduced BMI into the evaluation of fat deposition trait levels in Hu sheep, and revealed the different microbial and functional pathways in the gastrointestinal tract of Hu sheep with different BMI levels. We found significant positive correlation between BMI and fat deposition traits in Hu sheep. Among the gastrointestinal tracts of Hu sheep with different BMI levels, cecal microbial composition and function presented the greatest differences, which suggested that the cecum might be a key site for differences in BMI. Among the differential microbes in the cecum of the two groups, *Colidextribacter*, *Alloprevotella*, *Succenivibrio*, and *Lachnospiraceae_ND3007 Group* were significantly associated with BMI and 80% fat deposition traits in Hu sheep, which can be used as biomarkers affecting BMI in Hu sheep. The relationship between BMI and gastrointestinal microbiota provides a new direction for the regulation of fat deposition traits. In future work, we aim to integrate multiomics methods to study the specific functional pathways of different microbiota on fat deposition in Hu sheep, so as to provide a theoretical basis for the regulation of fat deposition traits.

## Figures and Tables

**Table 1:** Correlation analysis between BMI and fat deposition traits in sheep.

Characteristics	BMI
	Correlation
GR value, cm	0.37 **
Thickness of backfat, cm	0.23 **
Perirenal fat weight, kg	0.41 **
Relative weight of perirenal fat, %	0.29 **
Mesenteric fat weight, kg	0.47 **
Relative weight of mesenteric fat, %	0.33 **
Tail fat weight, kg	0.41 **
Relative weight of tail fat, %	0.14 **
Total fat weight, kg	0.54 **
Relative weight of total fat, %	0.33 **

** means *p* < 0.01.

**Table 2:** Description of fat traits and grouping difference test of high- and low-BMI Hu sheep.

Characteristics	All Hu Sheep (*n* = 357)	Low BMI Hu Sheep (*n* = 18)	High BMI Hu Sheep (*n* = 18)	*p*-Value
Body mass index, kg/m^2^	89.99 ± 8.36	74.26 ± 3.32	106.34 ± 2.93	<0.01
Body weight, kg	48.66 ± 7.23	37.08 ± 4.28	55.33 ± 4.91	<0.01
Body length, cm	73.38 ± 3.87	70.56 ± 3.42	72.06 ± 2.48	0.14
GR value, cm	2.61 ± 0.52	1.95 ± 0.40	2.86 ± 0.63	<0.01
Thickness of backfat, cm	0.63 ± 0.24	0.36 ± 0.12	0.75 ± 0.43	<0.01
Perirenal fat weight, kg	0.69 ± 0.36	0.37 ± 0.17	0.87 ± 0.45	<0.01
Relative weight of perirenal fat, %	1.39 ± 0.66	0.94 ± 0.35	1.57 ± 0.78	<0.01
Mesenteric fat weight, kg	1.22 ± 0.49	0.79 ± 0.39	1.51 ± 0.49	<0.01
Relative weight of mesenteric fat, %	2.47 ± 0.86	2.07 ± 0.93	2.73 ± 0.72	<0.01
Tail fat weight, kg	1.56 ± 0.49	1.11 ± 0.54	1.87 ± 0.62	<0.01
Relative weight of tail fat, %	3.17 ± 0.85	2.91 ± 1.41	3.38 ± 1.00	0.13
Total fat weight, kg	3.48 ± 1.06	2.27 ± 0.91	4.25 ± 1.10	<0.01
Relative weight of Total fat, %	7.03 ± 1.69	5.91 ± 2.11	7.69 ± 1.59	<0.01

Statistical data expressed as mean ± standard deviation. *p* < 0.05 indicates statistical significance.

**Table 3:** Analysis of differences between MRPP groups.

Group	A	Observed Delta	Expected Delta	Significance
L1–L2	−0.0005	0.5305	0.5303	0.5090
W1–W2	0.0160	0.6165	0.6266	0.0030
B1–B2	0.0104	0.6158	0.6223	0.0170
Z11–Z12	0.0024	0.5789	0.5803	0.2320
S1–S2	−0.0032	0.6752	0.6730	0.7090
K1–K2	0.0056	0.5623	0.5654	0.1230
H1–H2	0.0101	0.6228	0.6292	0.1350
M1–M2	0.0482	0.4297	0.4514	0.0010
J1–J2	0.0251	0.4257	0.4367	0.0010
Z21–Z22	0.0091	0.5706	0.5758	0.0340

Observed delta represents size of intragroup differences, expected delta represents difference between groups; a value less than 0 represents that intergroup differences were less than intragroup differences, and significance < 0.05 represents that intergroup differences are significant.

**Table 4:** Significant differences in cecum microbiota of sheep with different BMI at genus level.

Taxonomic Name	Relative Abundance		*p* Value	Trend
	Low BMI	High BMI		
*Oscillospiraceae_UCG-005*	11.248%	14.814%	0.008	+
*Prevotella*	4.791%	2.114%	0.040	−
*Prevotellaceae_UCG-001*	3.833%	1.706%	0.005	−
*Succinivibrio*	0.059%	0.695%	0.005	+
*Fibrobacter*	1.774%	0.552%	0.008	−
*Saccharofermentans*	1.283%	0.543%	0.005	−
*Succiniclasticum*	0.177%	0.022%	0.017	−
*Lachnospiraceae_UCG-010*	0.442%	0.724%	0.005	+
*Alloprevotella*	0.002%	0.153%	0.005	+
*Butyricicoccaceae_UCG-009*	0.458%	0.590%	0.022	+
*Methanocorpusculum*	0.124%	0.003%	0.005	−
*Agathobacter*	0.111%	0.297%	0.005	+
*Candidatus_Soleaferrea*	0.352%	0.497%	0.011	+
*Lachnospiraceae_NK3A20_group*	0.198%	0.046%	0.005	−
*Colidextribacter*	0.179%	0.420%	0.005	+
*Oscillibacter*	0.414%	0.542%	0.005	+
*Mogibacterium*	0.154%	0.053%	0.029	−
*Lachnospiraceae_UCG-002*	0.072%	0.146%	0.017	+
*Candidatus_Saccharimonas*	0.144%	0.049%	0.044	−
*Parabacteroides*	0.076%	0.178%	0.019	+
*Prevotellaceae_UCG-003*	0.048%	0.142%	0.005	+
*Family_XIII_AD3011_group*	0.218%	0.303%	0.014	+
*Lachnospiraceae_ND3007_group*	0.149%	0.006%	0.005	−
*Erysipelotrichaceae_UCG-009*	0.110%	0.050%	0.011	−
*[Ruminococcus]_gauvreauii_group*	0.113%	0.034%	0.005	−
*Dorea*	0.077%	0.114%	0.040	+

Species with an average relative abundance of less than 0.1% in both groups were excluded. Up-regulated bacteria in Hu sheep with high BMI shown with “+”; otherwise, they are shown with “−”.

## Data Availability

Raw data obtained by 16S rRNA sequencing were uploaded to the NCBI sequence read archive (submission ID: SUB10744410 and BioProject ID: PRJNA785332).

## Supplementary Materials

The following supporting information can be downloaded at: https://www.mdpi.com/article/10.3390/ani12070880/s1. Figure S1: rarefaction (left) and species accumulation curve (right) analysis the sequencing depth and sample size of different gastrointestinal samples; Figure S2: LEfSe analysis of different groups; Figure S3. enrichment analysis of functional pathways in different groups of level1 gastrointestinal tract; Figure S4: in different gastrointestinal tracts at level 1, there are significantly different functional pathways; Table S1: Overview of high-throughput sequencing data in different GI tract samples from Hu sheep; Table S2: analysis of alpha diversity in different parts of the gastrointestinal tract in BMI group.
