# Supplementary material for: Distribution and Difference of Gastrointestinal Flora in Sheep with Different Body Mass Index
Source: Animals (Basel). 2022 Mar 30;12(7):880. doi: 10.3390/ani12070880 (PMC8996880; doi:10.3390/ani12070880)
Supplement: Supplementary file 1 [file animals-12-00880-s001.zip › Supplementary Figure.pdf]

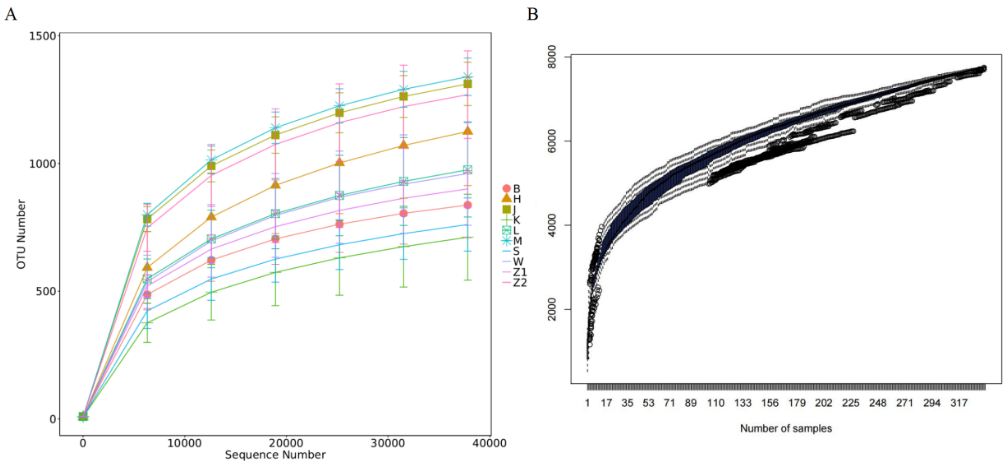

Figure S1. Rarefaction (A) and species accumulation curve (B) analysis the sequencing depth and sample size of different gastrointestinal samples.

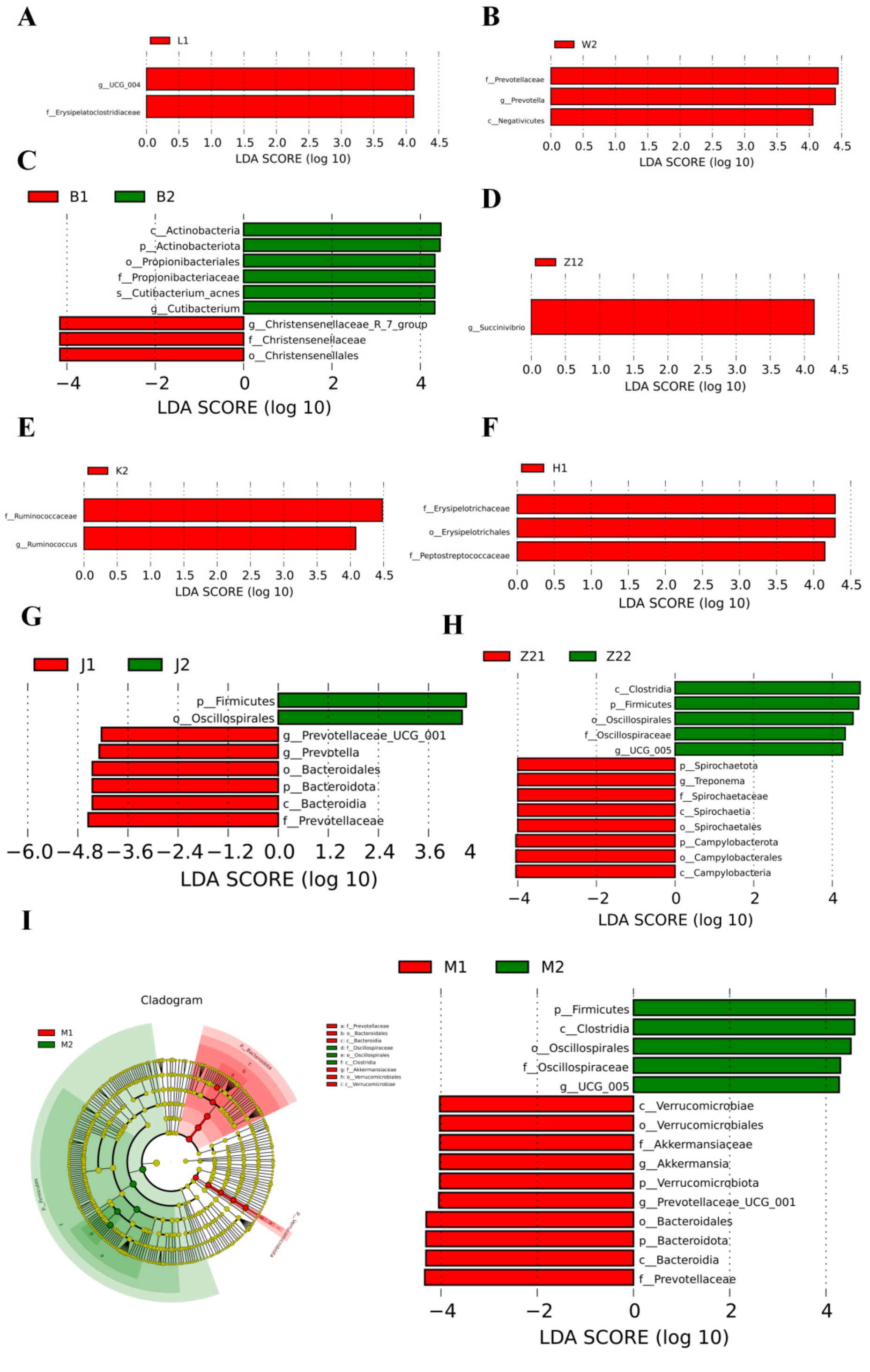

Figure S2. LEfSe analysis of different groups. (A) Rumen (B) Reticulum (C) Omasum (D) Abomasum (E) Jejunum (F) Ileum (G) Colon (H) Rectum (I) Cecum.

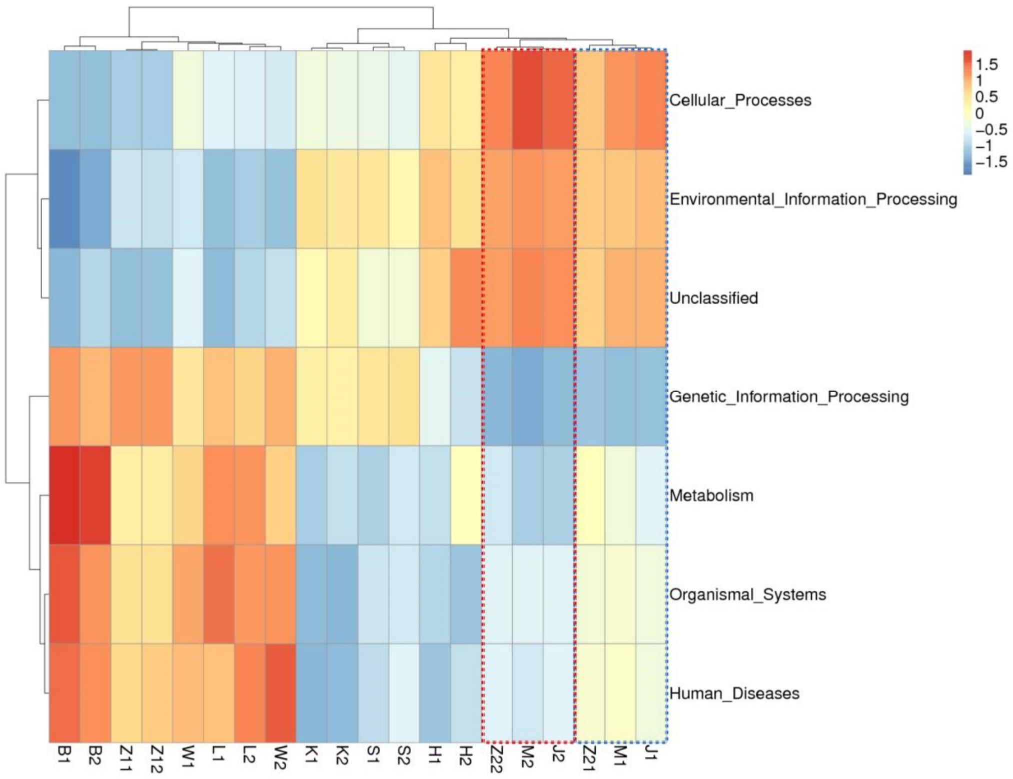

Figure S3. Enrichment analysis of functional pathways in different groups of level1 gastrointestinal tract.

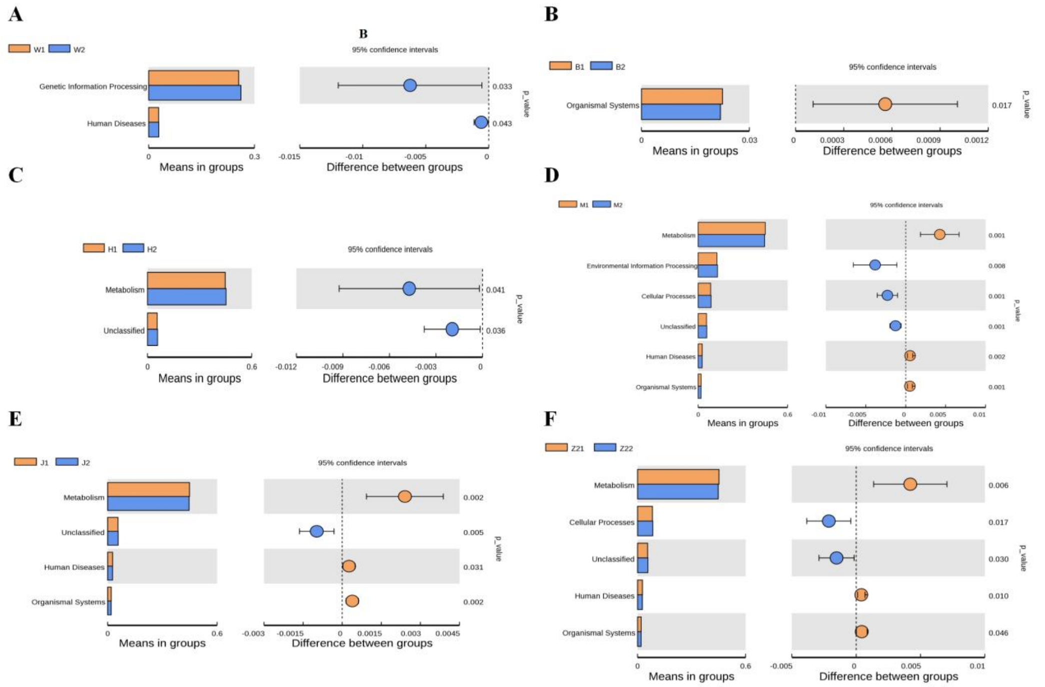

Figure S4. In different gastrointestinal tract, at level 1, there are significantly different functional pathway. (A) Reticulum (B) Omasum (C) Ileum (D) Cecum (E) Colon (F) Rectum.
