# Supplementary material for: Distribution and Difference of Gastrointestinal Flora in Sheep with Different Body Mass Index
Source: Animals (Basel). 2022 Mar 30;12(7):880. doi: 10.3390/ani12070880 (PMC8996880; doi:10.3390/ani12070880)
Supplement: Supplementary file 1 [file animals-12-00880-s001.zip › Supplementary Table.pdf]

Table S1. Overview of high-throughput sequencing data in different GI tract samples from Hu sheep.

Sample	Raw PE	Raw Tags	Clean Tags	Effectives Tags
L.9.1	82,815	81,782	80,072	64,108
L.22.2	84,429	82,885	81,295	64,934
L.14.1	85,380	83,493	80,777	63,444
L.26.2	93,746	92,625	90,237	60,890
L.16.1	84,477	83,102	81,491	63,011
L.8.1	93,822	92,177	89,491	63,282
L.24.2	95,448	94,972	93,724	69,327
L.18.1	80,270	79,848	78,821	69,053
L.25.2	85,824	85,406	84,270	65,256
L.21.2	90,497	89,402	87,460	61,338
L.5.1	82,688	78,790	76,087	59,754
L.15.1	97,259	95,139	92,717	64,594
L.28.2	86,977	84,973	82,691	68,227
L.36.2	86,880	85,474	83,570	65,081
L.3.1	92,384	90,377	88,373	68,232
L.2.1	95,278	93,339	91,423	61,613
L.32.2	96,300	94,133	92,424	64,257
L.33.2	92,544	88,799	86,444	68,833
L.34.2	81,360	79,660	78,000	64,202
L.20.2	92,627	90,797	89,104	63,387
L.31.2	89,778	87,413	85,488	66,685
L.23.2	87,346	85,321	83,608	65,262
L.7.1	99,331	96,403	93,755	62,197
L.11.1	80,038	77,657	75,934	65,867
L.17.1	85,975	83,984	81,656	65,433
L.1.1	97,365	94,348	91,981	61,747
L.13.1	98,922	96,988	94,836	66,313
L.29.2	91,876	82,566	80,646	69,474
L.12.1	89,020	87,294	85,387	69,555
L.10.1	93,215	91,198	89,239	60,879
L.35.2	84,864	83,358	81,483	67,210
L.30.2	87,268	85,640	83,917	63,181
L.4.1	91,358	89,411	87,195	65,878
L.27.2	89,877	88,079	86,290	67,609
L.6.1	81,070	79,866	78,231	68,868
L.19.2	73,137	71,827	70,414	59,963
W.1.1	100,400	84,156	81,409	59,784
W.2.1	108,696	95,872	92,418	64,398
W.3.1	100,176	84,438	81,346	59,978
W.4.1	101,624	89,507	86,182	61,401
W.5.1	81,809	69,397	67,485	52,237
W.6.1	107,402	94,496	91,675	67,986

W.7.1	110,682	89,932	86,192	65,798
W.8.1	96,381	84,912	81,935	59,605
W.9.1	100,763	85,198	82,092	61,872
W.10.1	99,186	82,160	79,060	58,572
W.11.1	100,271	82,228	79,226	61,543
W.12.1	93,106	76,991	74,427	59,940
W.13.1	82,527	62,244	60,019	51,623
W.14.1	90,397	71,703	69,137	54,629
W.15.1	94,824	81,789	79,129	58,681
W.16.1	81,570	68,141	66,017	52,838
W.17.1	98,887	87,442	84,276	58,070
W.18.1	98,978	83,528	80,473	60,443
W.19.2	91,825	78,474	76,122	56,124
W.20.2	85,114	69,780	67,503	50,836
W.21.2	101,843	90,648	87,722	60,393
W.22.2	84,291	67,528	64,946	51,161
W.23.2	98,026	84,545	81,936	61,398
W.24.2	101,542	87,662	84,729	65,707
W.25.2	99,197	86,776	83,391	60,563
W.26.2	95,705	79,931	77,355	55,785
W.27.2	103,342	89,399	85,500	62,896
W.28.2	107,134	93,502	89,350	63,394
W.29.2	105,828	94,271	90,871	66,687
W.31.2	73,454	62,992	61,202	47,505
W.33.2	100,314	87,544	84,287	64,353
W.34.2	96,553	83,786	80,923	58,586
W.35.2	80,517	70,028	68,038	49,955
W.36.2	90,633	74,590	71,749	52,999
W.30.2	84,283	66,823	63,923	49,734
W.32.2	108,804	96,631	93,353	67,418
B.1.1	80,444	68,561	66,451	51,847
B.2.1	73,982	54,531	52,429	46,464
B.3.1	98,764	86,543	83,692	62,255
B.4.1	90,096	77,942	75,361	52,335
B.6.1	96,446	83,942	81,385	62,559
B.7.1	84,938	72,317	69,989	52,432
B.8.1	97,127	83,148	80,416	57,209
B.10.1	94,031	78,911	75,985	57,797
B.11.1	87,807	76,230	73,859	55,009
B.12.1	82,065	65,971	63,498	49,350
B.13.1	83,609	72,376	70,318	53,696
B.14.1	79,635	61,224	58,853	45,589
B.15.1	99,732	86,846	83,264	60,347
B.16.1	85,994	75,253	73,193	54,395

B.18.1	97,851	84,670	81,756	60,017
B.19.2	86,700	74,689	72,434	53,823
B.20.2	92,789	79,973	77,550	55,262
B.22.2	100,529	86,322	83,019	59,871
B.24.2	86,127	70,585	68,138	52,935
B.25.2	98,976	86,341	83,634	60,555
B.26.2	75,226	59,091	57,039	47,962
B.27.2	87,090	77,134	74,397	52,946
B.28.2	88,742	76,825	74,375	56,791
B.30.2	85,924	72,686	70,218	50,307
B.31.2	86,906	74,404	72,204	55,761
B.32.2	107,558	94,043	90,588	67,279
B.33.2	92,252	80,081	77,527	56,944
B.34.2	92,946	81,213	78,540	57,171
B.35.2	89,145	78,105	75,839	53,012
B.36.2	95,594	81,554	79,128	62,125
B.9.1	98,648	89,543	86,385	61,014
B.17.1	97,505	86,164	83,053	62,215
B.21.2	90,579	76,751	73,887	57,021
B.23.2	92,560	78,705	75,959	57,317
B.29.2	112,068	100,119	97,139	66,606
B.5.1	103,304	101,533	99,266	62,316
Z1.1.1	102,305	87,772	85,056	60,372
Z1.2.1	89,710	70,162	67,443	55,175
Z1.3.1	84,635	73,599	71,542	53,941
Z1.4.1	92,400	81,529	79,230	55,296
Z1.5.1	100,497	86,818	84,170	61,612
Z1.6.1	106,117	92,830	90,002	67,291
Z1.7.1	98,565	84,841	82,496	63,432
Z1.8.1	102,986	89,644	86,559	63,917
Z1.9.1	100,667	87,458	84,840	60,303
Z1.10.1	95,116	83,891	81,574	61,366
Z1.11.1	101,601	92,060	89,290	63,871
Z1.12.1	89,994	78,836	76,388	56,780
Z1.13.1	74,348	64,755	63,092	48,219
Z1.14.1	98,761	85,157	82,431	63,136
Z1.15.1	102,707	93,160	89,908	63,846
Z1.16.1	90,930	82,181	79,332	57,898
Z1.17.1	78,331	69,664	67,661	49,336
Z1.18.1	88,565	78,936	76,646	54,969
Z1.19.2	90,919	81,658	79,340	52,764
Z1.20.2	89,407	80,356	78,204	54,835
Z1.21.2	84,398	74,297	72,153	53,607
Z1.22.2	92,183	83,280	80,821	54,793

Z1.23.2	110,422	98,857	95,493	65,371
Z1.24.2	84,479	72,658	70,545	54,503
Z1.25.2	89,773	78,903	78,278	57,321
Z1.26.2	101,180	88,277	85,576	63,651
Z1.27.2	88,362	79,507	77,298	55,521
Z1.28.2	96,111	86,673	83,912	60,656
Z1.29.2	97,966	88,704	86,228	61,202
Z1.30.2	95,141	84,533	81,832	59,520
Z1.31.2	87,335	75,362	73,214	56,304
Z1.32.2	93,266	83,277	80,932	60,049
Z1.33.2	99,338	89,381	86,856	59,611
Z1.36.2	98,272	96,028	93,457	61,140
Z1.34.2	99,493	97,379	95,040	59,993
Z1.35.2	108,225	106,370	104,175	67,477
S.1.1	95,824	94,408	92,986	60,896
S.2.1	107,518	105,395	101,696	66,126
S.3.1	108,421	106,902	105,201	67,718
S.4.1	103,784	101,905	99,757	61,752
S.5.1	105,278	103,397	101,499	63,654
S.6.1	104,140	102,728	101,268	65,896
S.7.1	106,032	104,415	102,766	62,431
S.8.1	100,007	98,629	96,797	69,193
S.9.1	99,246	97,714	96,019	60,519
S.10.1	111,097	109,403	107,848	66,095
S.11.1	95,989	94,427	92,482	61,844
S.12.1	99,304	97,980	96,359	63,708
S.13.1	101,213	99,370	97,600	63,250
S.14.1	110,010	108,570	106,603	67,791
S.15.1	109,532	107,761	106,156	67,775
S.16.1	100,280	98,824	96,855	60,111
S.17.1	99,184	97,333	95,016	61,662
S.18.1	107,853	106,099	104,262	66,165
S.19.2	115,655	114,010	112,061	69,687
S.20.2	101,654	100,021	98,317	62,275
S.21.2	107,871	106,155	104,350	65,639
S.22.2	100,795	99,359	97,680	62,103
S.23.2	106,527	104,223	101,390	64,544
S.24.2	101,541	100,193	98,521	66,594
S.25.2	107,810	106,166	104,047	68,528
S.26.2	102,110	100,574	98,884	63,789
S.27.2	106,584	105,117	103,483	65,188
S.28.2	103,957	102,057	99,745	67,232
S.29.2	96,486	94,777	92,904	62,464
S.30.2	105,486	104,191	102,584	65,199

S.31.2	102,540	101,222	99,542	64,580
S.32.2	101,786	99,977	97,688	63,457
S.33.2	99,673	98,271	96,639	64,517
S.34.2	104,910	103,586	101,985	62,303
S.35.2	98,372	96,642	94,822	60,948
S.36.2	104,537	102,945	101,330	61,999
K.1.1	113,595	111,703	109,791	69,548
K.2.1	110,583	109,061	106,924	69,165
K.3.1	108,250	106,970	105,425	69,316
K.4.1	101,978	100,324	98,551	64,420
K.5.1	102,473	100,908	99,163	64,702
K.6.1	106,357	105,013	103,337	68,381
K.7.1	104,481	102,875	101,147	65,305
K.8.1	103,897	102,310	100,604	66,351
K.9.1	95,679	94,155	92,529	61,891
K.10.1	108,011	106,794	105,357	68,798
K.11.1	95,864	93,510	91,151	61,182
K.12.1	99,994	98,191	96,452	60,851
K.13.1	107,789	105,371	103,286	68,190
K.14.1	103,390	101,442	98,965	66,580
K.15.1	104,165	102,289	100,301	65,026
K.16.1	101,913	100,050	98,154	65,853
K.17.1	96,854	94,621	92,347	62,386
K.18.1	98,353	96,029	94,065	63,319
K.19.2	99,473	97,588	95,507	61,862
K.20.2	97,743	95,771	94,075	61,857
K.21.2	95,434	93,733	92,002	61,617
K.22.2	97,876	96,226	94,544	62,746
K.23.2	103,610	100,994	98,811	63,120
K.24.2	108,967	106,897	105,111	65,853
K.25.2	103,203	100,733	98,505	63,025
K.26.2	97,798	96,287	94,646	61,788
K.27.2	101,127	99,381	97,787	62,945
K.28.2	106,365	104,576	102,660	65,061
K.29.2	106,633	104,717	102,854	63,024
K.30.2	102,810	100,952	99,106	65,937
K.31.2	100,873	98,958	97,034	68,636
K.32.2	108,159	106,332	104,619	68,928
K.33.2	96,451	94,797	93,149	62,423
K.34.2	103,132	101,467	99,841	64,168
K.35.2	104,204	102,135	99,888	65,067
K.36.2	105,260	103,540	101,523	63,830
H.2.1	99,272	97,358	95,097	61,227
H.3.1	103,554	101,967	100,260	66,460

H.4.1	105,758	104,222	102,533	62,808
H.6.1	107,194	105,514	103,349	68,892
H.7.1	106,293	104,572	102,917	67,535
H.8.1	101,557	100,114	98,521	66,327
H.10.1	94,232	92,485	90,907	60,281
H.11.1	108,043	106,328	104,494	67,260
H.12.1	104,957	102,869	100,897	64,121
H.15.1	103,249	101,761	100,113	61,603
H.16.1	98,797	97,048	95,127	61,916
H.17.1	108,829	107,059	105,307	66,846
H.18.1	101,738	99,743	97,845	68,338
H.19.2	99,061	97,380	95,564	63,590
H.20.2	108,379	106,874	105,217	69,163
H.22.2	103,098	100,518	98,280	66,702
H.23.2	96,517	94,974	93,355	60,641
H.24.2	105,998	104,317	102,438	63,321
H.26.2	102,427	100,912	99,394	63,505
H.27.2	113,863	112,193	110,095	69,171
H.32.2	110,025	94,034	91,532	66,462
H.34.2	89,718	79,967	78,089	56,159
H.35.2	93,678	82,423	80,284	60,254
H.28.2	97,702	86,325	84,236	60,732
H.29.2	103,843	87,039	84,391	65,723
H.31.2	104,086	94,900	92,313	63,681
M.1.1	88,579	72,495	70,210	55,950
M.2.1	96,756	80,368	77,591	56,761
M.3.1	88,992	72,919	70,852	56,695
M.4.1	83,291	65,934	63,920	52,723
M.5.1	98,135	80,589	78,132	57,924
M.6.1	94,181	82,244	80,220	57,954
M.7.1	101,821	83,488	80,998	64,147
M.9.1	81,084	68,495	66,628	50,921
M.10.1	94,509	85,462	83,550	59,603
M.11.1	106,066	95,023	92,174	65,791
M.12.1	98,349	89,126	86,645	62,425
M.13.1	86,823	75,537	73,561	54,598
M.14.1	99,950	88,573	85,979	59,191
M.15.1	80,276	68,708	67,123	51,344
M.16.1	94,760	84,108	81,959	56,945
M.17.1	98,819	86,400	84,014	57,770
M.18.1	94,642	81,904	79,591	55,153
M.19.2	99,818	97,839	95,164	62,221
M.20.2	103,367	101,482	99,132	65,063
M.21.2	107,556	105,548	102,810	65,565

M.22.2	97,660	95,853	93,348	61,163
M.23.2	105,212	103,478	100,993	67,145
M.24.2	102,581	100,788	98,178	63,657
M.25.2	103,143	101,391	99,011	63,358
M.26.2	104,137	102,284	99,847	62,878
M.27.2	97,833	96,190	93,602	61,591
M.28.2	105,309	103,606	100,915	62,505
M.29.2	104,870	103,294	101,003	65,169
M.30.2	107,973	105,875	102,881	64,750
M.31.2	115,198	113,215	110,170	69,455
M.32.2	101,803	99,828	97,103	64,736
M.33.2	102,375	100,469	97,723	64,969
M.34.2	104,707	102,890	100,184	64,730
M.35.2	103,882	101,857	98,863	62,513
M.36.2	104,504	102,540	99,813	66,435
M.8.1	105,250	97,027	93,882	63,388
J.1.1	101,130	99,417	96,877	64,507
J.2.1	103,523	101,559	98,701	61,219
J.3.1	102,122	100,315	97,536	65,228
J.4.1	100,604	98,694	95,737	67,645
J.5.1	104,458	102,768	100,115	66,320
J.6.1	105,940	103,773	100,648	65,737
J.7.1	113,783	111,590	108,689	69,849
J.8.1	112,813	110,288	106,753	69,243
J.9.1	95,236	93,356	90,851	60,731
J.10.1	102,567	100,771	97,869	62,834
J.11.1	108,277	106,288	103,225	65,593
J.12.1	104,401	102,358	99,457	63,867
J.13.1	109,657	107,619	104,508	67,511
J.14.1	107,810	105,659	102,596	67,457
J.15.1	96,261	94,660	92,304	61,813
J.16.1	99,351	97,502	94,639	61,155
J.17.1	103,073	101,313	98,951	63,472
J.18.1	94,256	92,224	89,635	60,695
J.19.2	101,276	99,007	96,372	60,954
J.20.2	107,553	105,476	102,427	66,926
J.21.2	107,304	105,426	102,715	66,449
J.22.2	98,130	96,284	93,551	62,691
J.23.2	100,073	97,883	94,705	65,479
J.24.2	101,548	99,657	96,892	61,557
J.25.2	100,774	98,545	95,579	61,766
J.26.2	96,887	95,181	92,632	62,322
J.27.2	104,253	102,393	99,667	64,952
J.28.2	104,558	102,897	100,397	62,819

J.29.2	97,831	96,304	94,066	63,561
J.30.2	97,287	84,222	81,689	60,487
J.31.2	104,692	94,988	92,361	62,370
J.32.2	96,959	85,548	83,375	58,808
J.33.2	91,165	83,721	81,534	56,526
J.34.2	97,296	88,901	86,340	61,784
J.35.2	102,410	92,344	89,604	63,666
J.36.2	105,559	94,982	92,345	64,553
Z2.2.1	114,724	103,049	99,544	68,353
Z2.3.1	102,200	92,798	90,356	62,714
Z2.4.1	101,681	92,344	89,428	65,756
Z2.5.1	101,752	92,210	89,547	63,429
Z2.7.1	105,776	95,195	92,042	62,470
Z2.8.1	100,947	87,238	84,593	63,113
Z2.9.1	102,514	93,198	90,706	64,607
Z2.10.1	99,834	89,235	86,894	63,729
Z2.12.1	102,862	92,623	89,410	62,859
Z2.14.1	103,842	93,598	90,387	61,637
Z2.15.1	102,857	92,577	90,138	65,787
Z2.16.1	96,665	85,389	82,934	62,654
Z2.17.1	105,414	95,320	92,461	67,000
Z2.20.2	100,341	90,272	87,879	63,928
Z2.22.2	100,989	91,793	88,886	62,810
Z2.23.2	101,690	92,390	89,676	65,182
Z2.24.2	106,069	96,063	93,112	64,325
Z2.26.2	91,442	84,281	82,005	53,810
Z2.28.2	76,701	71,160	69,392	46,950
Z2.29.2	84,002	70,323	68,418	52,315
Z2.30.2	100,439	89,927	87,629	63,119
Z2.32.2	103,199	88,944	86,326	61,846
Z2.33.2	87,741	79,810	77,908	55,764
Z2.34.2	75,249	67,683	66,130	47,215
Z2.35.2	65,804	59,616	58,180	40,532
Total	33,143,103	30,842,807	30,051,711	20,928,585

Table S2. Analysis of alpha diversity in different parts of gastrointestinal tract in BMI group.

Items		observed species	Shannon	Chao1
	Low	929.44±173.24	6.82±0.47	1136.59±236.79
Rumen	High	1020.50±194.53	6.97±0.47	1255.22±258.58
	P	0.11	0.32	0.10
	Low	1034.67±235.83	6.34±0.61	1252.01±289.92
Reticulum	High	886.33±141.09	6.66±0.42	1019.61±168.18
	P	0.06	0.05	0.01
	Low	803.22±126.20	6.31±0.51	922.95±176.69
Omasum	High	870.94±127.48	6.54±0.76	983.41±172.35
	P	0.03	0.04	0.12
	Low	903.06±87.03	6.65±0.70	1049.21±111.61
Abomasum	High	897.44±57.85	6.54±0.49	1050.90±93.18
	P	0.63	0.39	0.91
	Low	746.89±83.38	5.54±0.91	920.80±106.96
Duodenum	High	774.83±125.14	5.64±1.11	959.24±148.58
	P	0.79	0.63	0.65
	Low	658.06±96.65	4.88±0.88	827.47±133.74
Jejunum	High	764.00±211.22	5.40±0.56	974.06±275.13
	P	0.09	0.07	0.80
	Low	1157.69±178.07	5.99±1.00	1448.61±197.05
Ileum	High	1092.62±251.09	5.94±1.41	1316.36±259.57
	P	0.45	0.92	0.11
	Low	1340.17±98.64	7.80±0.26	1496.89±131.62
Cecum	High	1338.39±41.33	8.08±0.25	1572.67±77.22
	P	0.42	<0.01	0.01
	Low	1278.56±46.15	7.84±0.26	1513.30±63.14
Colon	High	1344.39±103.79	8.00±0.28	1560.14±132.55
	P	0.09	0.09	0.31
	Low	1317.54±156.36	7.72±0.47	1560.03±205.77
Rectum	High	1217.00±185.15	7.74±0.38	1382.53±243.73
	P	0.17	0.61	0.06

* Statistical data are expressed as mean ± standard deviation. $P < 0.05$ indicates statistical significance.
